# Collision thyroid tumors: a case report of coexisting papillary and follicular carcinoma

**DOI:** 10.1093/jscr/rjaf1106

**Published:** 2026-01-20

**Authors:** Danilo Estuardo Torselli-Valladares, Víctor Argueta, Carlos Rene Cordón, Marco Antonio Peñalonzo

**Affiliations:** Universidad Francisco Marroquin, Faculty of Medicine, 6 avenue 7-55 zona 10, Guatemala 01010, Guatemala; Hospital General San Juan de Dios, 1A calle 10-50 zona 1, Guatemala 01010, Guatemala; Hospital General San Juan de Dios, 1A calle 10-50 zona 1, Guatemala 01010, Guatemala; Universidad Francisco Marroquin, Faculty of Medicine, 6 avenue 7-55 zona 10, Guatemala 01010, Guatemala; Universidad Francisco Marroquin, Faculty of Medicine, 6 avenue 7-55 zona 10, Guatemala 01010, Guatemala

**Keywords:** thyroid cancer, collision tumors, tumoral coexistence

## Abstract

Collision tumors of the thyroid gland are defined by the coexistence of two or more histologically and morphologically distinct neoplasms, separated by normal thyroid tissue. This phenomenon is rare, with the most frequently reported combination being the coexistence of medullary carcinoma and papillary carcinoma. This case reports a 73-year-old female patient with an asymptomatic thyroid nodule. Ultrasonographic examination revealed a solid, isoechoic, well-defined nodule with a halo and peripheral vascularization, measuring 4.3 × 2.5 cm in the left lobe. Fine-needle aspiration cytology classified the nodule as Bethesda IV (follicular tumor). Following total thyroidectomy, histopathological analysis identified papillary carcinoma in the isthmus and minimally invasive follicular carcinoma in the left lobe.

## Introduction

The coexistence of cancers of different lineages within the thyroid gland is an extremely rare finding. The term “collision tumors” refers to the simultaneous presence of two or more histologically and morphologically distinct tumors, or metastases from other organs, separated by normal thyroid tissue [1–3].

Synchronous thyroid carcinomas are classified into three types: mixed tumors, which present a combined cellular population of common origin; composite tumors, which contain two distinct cellular populations within the same tumor; and collision tumors [1, 2]. All these types are exceedingly rare and account for <1% of thyroid cancers [1].

The most common association is between medullary carcinoma and papillary carcinoma, followed by metastatic tumors and papillary carcinoma. The coexistence of papillary carcinoma with follicular carcinoma is the most unusual [1–5].

## Case presentation

A 73-year-old female patient consulted for a thyroid nodule detected through self-palpation. She had no associated symptoms, and normal thyroid function. Her medical history included type II diabetes and hypertension, both under control, as well as idiopathic thrombocytopenia in remission for 1 year.

**Figure 1:**
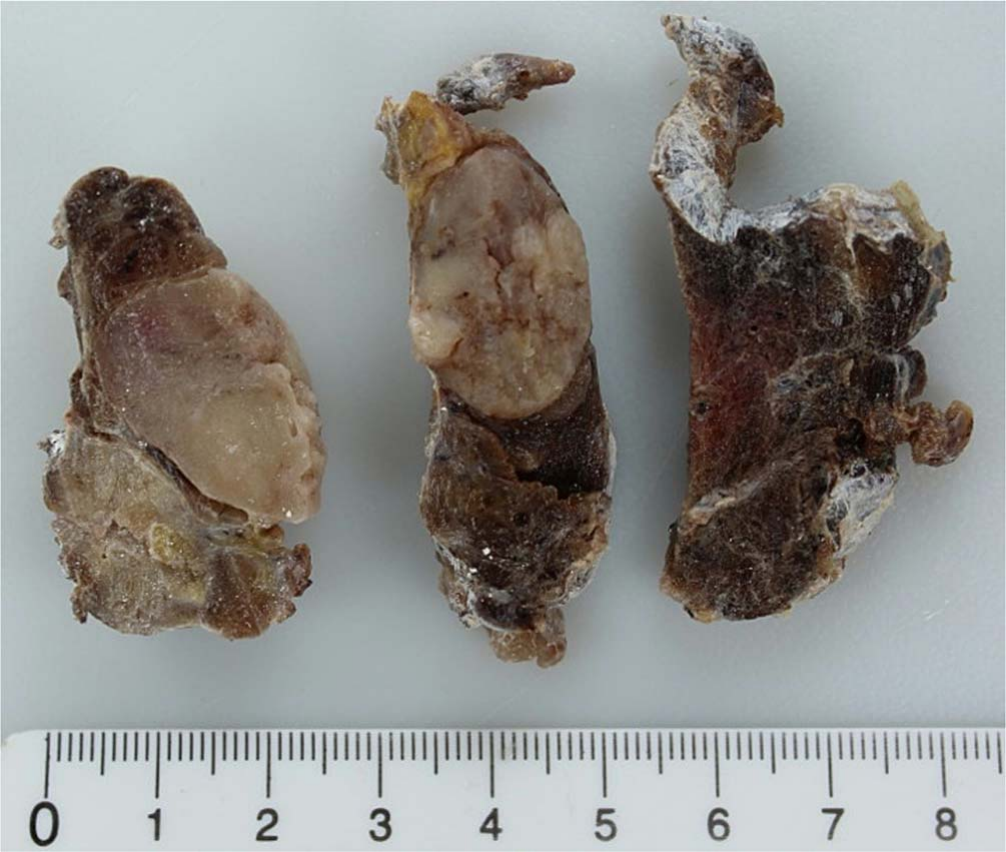
Sections of the left thyroid lobe and isthmus showing follicular neoplasia. A papillary carcinoma is visible in the lower part of the first section.

Ultrasound revealed an isoechoic nodule in the left lobe, well-defined, with a complete peripheral halo and peripheral vascularization, measuring 4.3 × 2.5 cm. Fine-needle aspiration classified the nodule as a follicular tumor, Bethesda category IV. A left lobectomy with isthmectomy was planned. However, during the procedure, a second nodule measuring 1.5 cm was discovered in the isthmus, macroscopically suggestive of papillary carcinoma. As a result, a total thyroidectomy was performed (Fig. 1).

**Figure 2:**
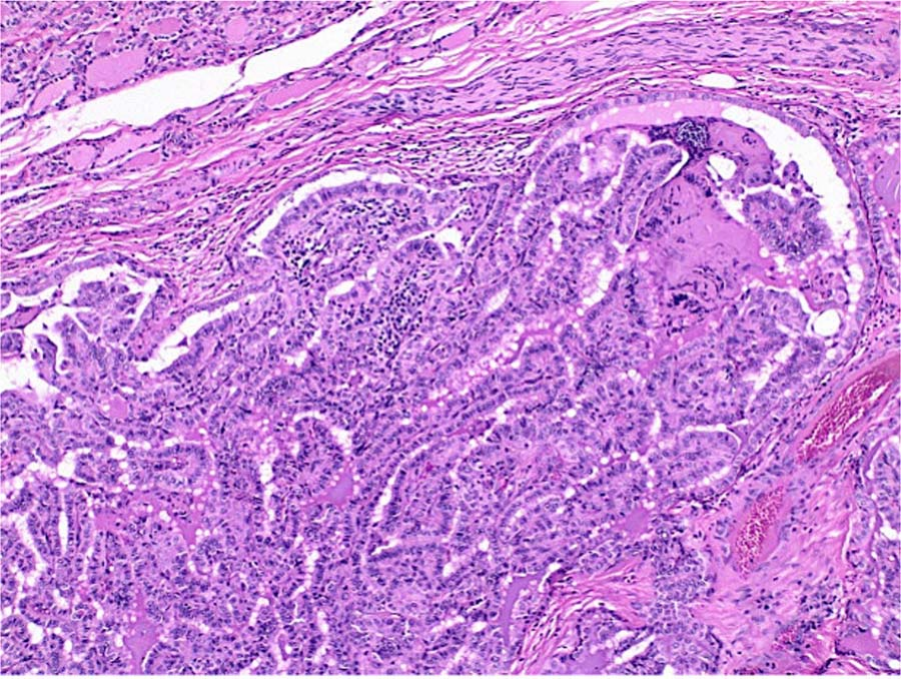
Histological section of papillary carcinoma showing papillary architecture, clear nuclei, and nuclear membrane thickening.

**Figure 3:**
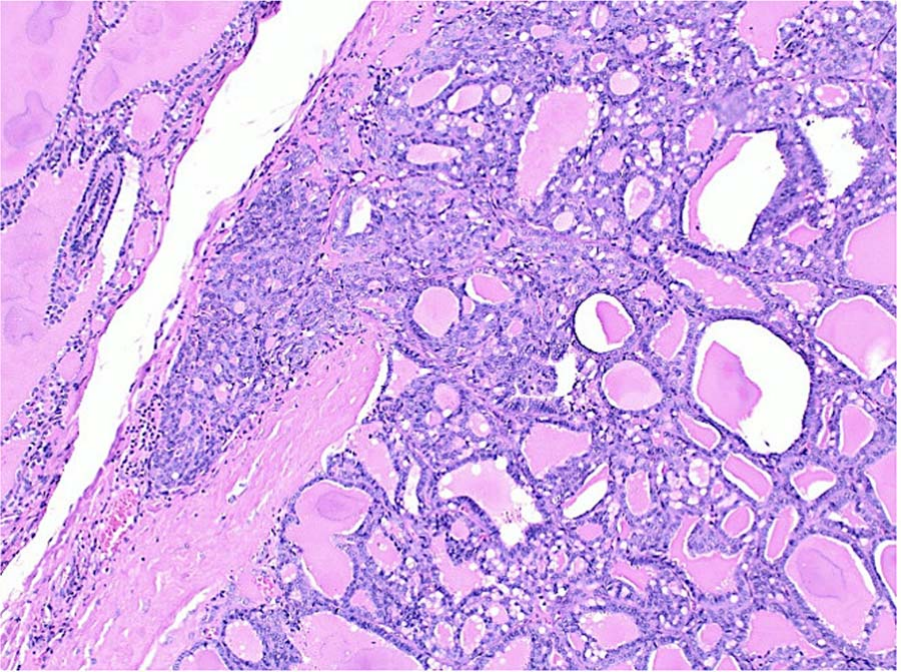
Histological section of follicular carcinoma demonstrating capsular invasion through its full thickness.

**Figure 4:**
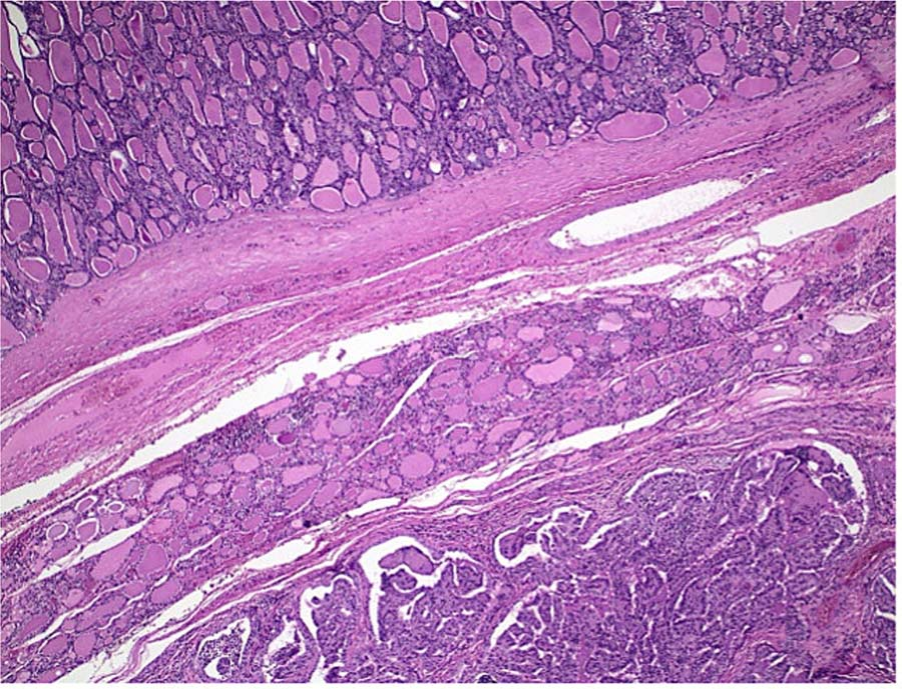
Histological section showing follicular carcinoma in the upper region and papillary carcinoma in the lower region, with intervening non-neoplastic thyroid tissue.

The final pathological report revealed a 1.4 cm classic papillary carcinoma in the isthmus, with extrathyroidal extension, without vascular, lymphatic, or perineural invasion, and with clear margins. In the left lobe, a minimally invasive follicular carcinoma of 3.5 cm was diagnosed, with capsular and limited vascular invasion (less than four vessels), without lymphatic or perineural invasion, and with clear margins (Figs 2–4).

The patient experienced no postoperative complications and was referred for a therapeutic dose of 100 mCi of radioactive iodine as additional treatment.

Following the total thyroidectomy and adjuvant radioactive iodine treatment, therapeutic decisions were guided by the dominant tumor, which in this case was the follicular thyroid carcinoma. The patient was started on levothyroxine suppression therapy at 1.5 mcg/kg/day, targeting low-normal Thyroid-stimulating hormone (TSH) levels. She was subsequently enrolled in an active surveillance program, with evaluation every 6–12 months with serum TSH, free thyroid hormones, thyroglobulin, and anti-thyroglobulin antibodies, as well neck ultrasound. To date, the patient has shown no biochemical or structural evidence of recurrence, with appropriate adherence to therapy.

## Discussion

The multicentricity of well-differentiated thyroid cancers is no longer considered unusual, but the simultaneous presence of two histologically distinct malignant tumors is an exceptional event. To classify tumors as collision tumors, three criteria must be met: (1) each tumor must exhibit a clear malignancy pattern, (2) each must be histologically and morphologically distinct, and (3) it must be ruled out that one is a metastasis of the other [4]. In this case, the tumors meet these criteria, justifying their classification as collision tumors.

The prevalence of these tumors is difficult to determine. In a review of 395 thyroid cancer cases, three (0.9%) were diagnosed as collision tumors [5]. In a study conducted in India involving 138 patients who underwent surgery, five cases (3.6%) were identified [4]. The most common combinations are medullary carcinoma with papillary carcinoma and squamous carcinoma with papillary carcinoma [6]. The coexistence of papillary carcinoma and follicular carcinoma is rare, with the first documented case reported in 2013 by Plauche [2], and by mid-2023, a total of 36 cases had been recorded [4]. This coexistence is attributed to the fact that papillary carcinoma is the most common subtype and often presents as a microcarcinoma.

Suspecting the presence of collision tumors from a clinical standpoint is challenging. Most diagnoses are made after the surgical specimen is evaluated. In our case, after performing a lobectomy and isthmectomy, two distinct tumors were observed, leading to the completion of the total thyroidectomy during the same procedure.

The etiopathogenesis of these tumors is not clearly established, although several theories have been proposed. The first suggests that one tumor alters the microenvironment, affecting blood flow and oxygen availability, which may predispose the development of a second tumor. The second theory posits that both tumors arise from the same pluripotent stem cells, which may differentiate into different cellular lines. The third theory suggests that the appearance of distinct tumors may be a random event [4].

The prognosis, management, and follow-up of patients with collision thyroid tumors remain uncertain, as many publications are based on isolated cases or small series with limited follow-up. A multidisciplinary approach, guided by the more aggressive tumor, is recommended [6]. Some authors believe that these tumors should be evaluated and treated individually, while others consider them more aggressive and at greater risk of recurrence compared to independent tumors [7–9]. In this case, both are well-differentiated cancers, whose management is well established.

Molecular studies were not performed to determine the genetic profile of the tumors. However, the standard treatment for both papillary and follicular carcinomas is surgical resection, the extent of which depends on factors such as the patient's age and tumor size. Radioactive iodine is part of the adjuvant treatment, and the follow-up for both types of cancer does not vary significantly. In contrast, other types of collision tumors with distinct biological behaviors present more complex and controversial management [10, 11].

## Conclusion

In conclusion, thyroid collision tumors are rare and pose questions regarding their etiopathogenesis, biological behavior, treatment options, and long-term follow-up. This case highlights the challenges in preoperative identification and the need for a comprehensive surgical and pathological evaluation. While management follows established guidelines for well-differentiated thyroid carcinomas, collision tumors introduce additional complexities due to their uncertain prognosis.
